# Acute psychosocial stress and emotion regulation skills modulate empathic reactions to pain in others

**DOI:** 10.3389/fpsyg.2014.00517

**Published:** 2014-05-30

**Authors:** Gabriele Buruck, Johannes Wendsche, Marlen Melzer, Alexander Strobel, Denise Dörfel

**Affiliations:** ^1^Work and Organizational Psychology, Department of Psychology, Technische Universität DresdenDresden, Germany; ^2^Neurogenetics and Individual Differences, Department of Psychology, Technische Universität DresdenDresden, Germany; ^3^Division of Mind and Brain Research, Department of Psychiatry and Psychotherapy, Charité Medical University BerlinBerlin, Germany

**Keywords:** emotion regulation skills, acute psychosocial stress, empathy for pain, acceptance, tolerance, TSST

## Abstract

Psychosocial stress affects resources for adequate coping with environmental demands. A crucial question in this context is the extent to which acute psychosocial stressors impact empathy and emotion regulation. In the present study, 120 participants were randomly assigned to a control group vs. a group confronted with the Trier Social Stress Test (TSST), an established paradigm for the induction of acute psychosocial stress. Empathy for pain as a specific subgroup of empathy was assessed via pain intensity ratings during a pain-picture task. Self-reported emotion regulation skills were measured as predictors using an established questionnaire. Stressed individuals scored significantly lower on the appraisal of pain pictures. A regression model was chosen to find variables that further predict the pain ratings. These findings implicate that acute psychosocial stress might impair empathic processes to observed pain in another person and the ability to accept one's emotion additionally predicts the empathic reaction. Furthermore, the ability to tolerate negative emotions modulated the relation between stress and pain judgments, and thus influenced core cognitive-affective functions relevant for coping with environmental challenges. In conclusion, our study emphasizes the necessity of reducing negative emotions in terms of empathic distress when confronted with pain of another person under psychosocial stress, in order to be able to retain pro-social behavior.

## Introduction

Pain comprises manifold sensory, affective, and cognitive experiences that often mirror personal life events and depend on individual differences. Hence, for an observer wishing to understand a person's pain and to empathize with the other's feelings, this complexity constitutes a great challenge. Considering this, the social communications model of pain (Hadjistavropoulos and Craig, 2002) points to the importance of attending to both the sender of information and the receiver, thereby emphasizing the importance of individual differences on both the person suffering from pain and the person observing it. Although this model focuses on the communication of pain, it recognizes that emotion often is intermixed with the pain that is communicated. Hence, by observing another person's pain the affective state of this person is also perceived and may lead to a similar affective state in the observer (Craig et al., 2010). Following this, pain is a multidimensional phenomenon which provides a warning for the suffering person, but also a signal to attract the attention of others (Craig, 2006, 2009) in order to receive comfort, relief, or medical aid. Taking the observer perspective, the concept of pain, and the construct of empathy are overlapping, because to feel with another person in pain might constitute a prerequisite for initiating helping behavior.

“Empathy in the broadest sense refers to the reactions of one individual to the observed experiences of another” (Davis, 1983, p. 113). Singer and Lamm (2009) briefly describe empathy as an affective reaction to someone else's affective state. Walter (2012) characterizes affective empathy as (a) an affective state that is (b) elicited by the affective state of another; (c) is similar (isomorphic) to the other's affective state; (d) is oriented toward the other; and (e) includes perspective taking, Self-other distinction, and knowledge of the causal relation between one's own and the other's affective state. Hence, empathy constitutes a multifaceted construct containing both cognitive and affective processes (Davis, 1983, 2006) which contribute to empathic behavior and expression (Eisenberg, 2005; Walter, 2012; Leiberg and Singer, 2013). Empathy is not a mandatory reaction, but rather occurs when a motivation to act is triggered by the observation and the understanding of another person's feelings, especially negative feelings such as anxiety, depression, or pain. Observing another person in pain triggers empathic reactions very reliably (Singer et al., 2004; Botvinick et al., 2005; Jackson et al., 2005; Avenanti et al., 2006; Moriguchi et al., 2007; Lamm et al., 2008, 2011; Akitsuki and Decety, 2009; Decety, 2009; Craig et al., 2010; Hein et al., 2011), even without an own experience of that pain (Danziger et al., 2009). Hence, studying the perception of pain in others provides an efficient, well-established avenue for investigating human empathy.

Previous research concludes that empathic reactions to pain in others are generated by two distinct ways. A stimulus-response, perception-based route is triggered in the presence of concrete visual stimuli depicting, for example, other people or body parts in painful situations. Additionally, in situations where such direct perceptual evidence is missing, affective states of others can be inferred by the creation of representations of the other's potential mental state, which constitutes a more abstract, inferential route (Singer and Lamm, 2009; Walter, 2012; Engen and Singer, 2013). Thus, Singer et al. (2004) could demonstrate that empathic responses to the pain of another person could even be elicited automatically in the absence of an emotional cue (such as facial emotional expressions).

The perception-based route activates core empathy-related brain networks via simulation of the affective state observed through the engagement of action-perception networks (Preston and De Waal, 2002). The perception–action model posits that the observer resonates with the emotional state of another individual by activating the motor representations and associated autonomic and somatic responses that stem from the observed person. This perception–action coupling constitutes a crucial component in the neural architecture underlying empathy (Preston and De Waal, 2002; Decety and Jackson, 2004). In accordance with this, several studies concerning the neural correlates of empathy have focused on empathy for pain demonstrating shared neurophysiological mechanisms between the first-hand experience of pain, the perception of pain in others (Decety and Lamm, 2006; Simon et al., 2006; Lamm et al., 2011), and the evaluation of pain in others (Jackson et al., 2005, 2006a).

A partial neural overlap between the experience of pain in the Self and the observation of pain in others has been reported in the somatosensory cortex/posterior insula, which is associated with the sensory discriminative dimension of pain (Avenanti et al., 2005, 2006), the dorsal anterior cingulate cortex (dACC, often referred to as posterior ACC and extending into anterior middle cingulate cortex, aMCC), the thalamus, and the anterior insula (AI; Singer et al., 2004; Lamm et al., 2011). The dACC and the AI are involved in the affective aspects of pain processing stemming from interoceptive awareness and meta-representations of global emotional moments (Craig, 2009; Decety, 2011; Lamm et al., 2011).

However, as stated above, empathy includes perspective taking, Self-other distinction, and knowledge of the causal relation between one's own and the other's affective state (Walter, 2012). Imagining how another person feels and how one would feel oneself require distinct forms of perspective taking that carry different emotional consequences. The former may evoke empathic concern, while the latter induces both empathy and personal distress (Batson et al., 1997, 2003). “Indeed, in order for the subjective experience to be labeled empathy, the observer must recognize that the emotion she/he is experiencing is a response to the other's emotional state” (Lamm et al., 2008, p. 56). In the study by Lamm et al. (2008) behavioral measures and event-related fMRI were used to investigate the effects of perspective taking (“imagine other” vs. “imagine Self”) while participants observed the facial expression of pain. The authors report that empathic concern was considerably stronger when participants focused on the feelings of the other, whereas adopting the self-perspective led to stronger personal distress. Additionally, imagining pain in the other person was associated with more activity in right superior and right inferior parietal lobe, regions that are related to perspective taking and sense of agency (David et al., 2006; Powell et al., 2010; Seghier, 2013). Trait measures of empathy (Empathy Quotient, empathic concern, emotional contagion) were also correlated to brain activity in the left posterior/middle insula, and the dACC, brain regions involved in affective pain processing (Lamm et al., 2008). These findings support the hypothesis that the affective network of pain processing is specifically involved in the perception of pain in others (see also Singer et al., 2004). In another study, which used a similar perspective taking manipulation, color pictures showing right hands and right feet of people in painful and non-painful situations were applied (Jackson et al., 2006a). The authors demonstrate that imagining the Self and imagining the other in painful situations are both associated with activation of the pain-related neural network. However, while the self-perspective engaged the insula bilaterally, the other-perspective involved mainly the insula in the right hemisphere. This may emphasize that imagining the other in pain is related to the feeling of pain and its emotional awareness, which is associated only with the right insula computing a higher order metarepresentation of primary interoceptive activities (Craig, 2003). Similarly to Lamm et al. (2008), taking the perspective of the other was associated with greater activations in the right temporo-parietal region, which is known to play a crucial role in perspective taking (Decety and Sommerville, 2003; Meltzoff and Decety, 2003).

To summarize, empathy for pain does not rely on a full overlap between the Self and the other. In contrast, experiencing another person's pain or distress in the same way as one's own pain experience would lead to an “empathic over-arousal” (Eisenberg, 2000). Taking the perspective of the other would lead to empathic concern, an important instigator of helping behavior, whereas self-perspective increases personal distress, which might produce an egoistic motivation to reduce personal distress (Batson et al., 1997).

Hence, “the best response to others' distress may not be distress, but efforts to soothe that distress” (Decety and Lamm, 2006, p. 1156). People who experience the others' emotions intensely, especially negative emotions, are prone to personal distress, i.e., an aversive emotional reaction such as anxiety or discomfort based on the recognition of another's negative affective state. Emotional distress may be in conflict with the motivation to feel for the other person, because it shifts priorities to the Self and toward short-term goals (for instance, getting quick relief from a painful situation; Decety and Lamm, 2006) and might prevent helping behavior. According to this, Cheng et al. (2007) found that physicians, who are frequently confronted with patients in pain, show less activation in AI, dACC, and somatosensory cortex while viewing pictures with needles being inserted into different body parts. Instead, the experts activated brain regions associated with higher cognitive control, emotion regulation, and perspective taking such as the medial and superior prefrontal cortices and the temporo-parietal junction.

Following this, emotion regulation provides the opportunity to modulate our emotional experience and behavior. It refers to a set of different strategies by which individuals “influence which emotions we have, when we have them and how these emotions are experienced or expressed” (Gross, 1998; p. 224). For instance, by using reappraisal one is able to change the meaning of an emotional stimulus, thereby reducing its threat or personal relevance. In contrast, distraction guides attention away from the emotion eliciting stimulus, while expressive suppression modifies the behavioral or physiological response to an emotional stimulus. If changing the meaning of or increasing the distance to the emotional situation by those strategies is not possible, strategies that focus on tolerating an unpleasant situation might be more effective. Specifically, individual adaptation to pain is supported by acceptance skills, e.g., as taught in the acceptance-based pain management program (Mathias et al., 2013; Sturgeon and Zautra, 2013). This strategy has been discussed as an important resource in the course of coping with pain (McCracken and Eccleston, 2003). Berking (2010) has proposed an integrative model of Adaptive Coping with Emotions (ACE) as a theoretical framework for identifying treatment targets in interventions aimed at improving emotion regulation. Different skills are distinguished from one another: The first cluster of skills is classified as mindfulness-/acceptance-based, the second as change-oriented. As a change-oriented component, the authors describe the *acceptance of negative emotions.* When necessary, one can prevent the triggering of further (secondary) negative emotions as a consequence of non-acceptance, which would impair the regulation process of the “primary” emotion.

Research about the effects of stress on empathy (for pain) primarily focuses on problems associated with chronic stress in health care employees. In this sector, studies show that empathy is blunted by stressors such as high workload, exposure to suffering patients or patient death, and ethical conflicts (Koehl-Hackert et al., 2012; West, 2012; Newton, 2013). However, there is an obvious lack of experimental studies on the association between acute psychosocial stress and its influence on empathy and the observation of pain in others, which might be able to disentangle the mechanisms that contribute to the possibly detrimental effects of stress on empathy. To our knowledge, up to now only few studies addressed this issue. Smeets et al. (2009) report effects of cortisol elevations by a psychosocial stress task on social cognition. The authors found that in males a high cortisol response was associated with enhanced social cognition in a task for the assessment of mindreading abilities. In contrast, women with a low cortisol response were better in correctly inferring emotional mental states. This study not only highlights the impact of stress on cognitive and affective empathy, but also emphasizes the fact that an objective stress situation might not lead to negative effects *per se*. Kukolja et al. (2008) found an influence of elevated levels of the stress-related hormones (i.e., norepinephrine and cortisol) on amygdala responses to socio-emotional stimuli. Dedora et al. (2011) report that in an emotion-identification task, participants exposed to acute stress named emotions more rapidly than without acute stress. Unfortunately, the authors did not report psychological measures of variables potentially moderating the impact of acute stress on social cognition.

The influence of stress on empathy can partly be explained by stress-related effects on the prefrontal cortex because of its involvement in processes concerning emotion regulation, working memory, self-regulatory processes, and goal-directed behavior (Miller, 2000; Arnsten, 2009; McEwen and Morrison, 2013). These neural networks operate as top–down mediators that are crucial for regulating emotions inasmuch as they enhance flexible and appropriate responses to external and internal stimuli (Decety, 2011). The variety of potential emotional reactions to stressful situations may in part be explained by the application of different emotion regulation strategies. However, acute stress experiences may in turn also affect the application of emotion regulation strategies (Raio et al., 2013). Consequentially, empathic reactions to another person in pain are influenced by stress, especially in individuals exhibiting dysfunctional emotion regulation strategies (Decety, 2011).

Neurophysiological studies have repeatedly shown that voluntary top–down emotion regulation by different cognitive strategies rests upon executive control regulating (negative) emotions by an activation of brain regions like the PFC and the parietal cortex. These regulation processes modulate the emotional experience processed by bottom-up emotion processing structures, e.g., the amygdala (Ochsner et al., 2004; Ochsner and Gross, 2005; Kalisch, 2009; Walter et al., 2009; Erk et al., 2010). Importantly, individual differences in emotion regulation skills may alter the effectiveness of executive functions (Drabant et al., 2009; Abler et al., 2010; Webb et al., 2012). Additionally, situational demands such as the experience of acute stress impair the top–down control of emotions (Arnsten, 2009).

Supporting this, Decety and Meyer (2008) proposed a model that combines emotion regulation and empathy. The authors describe bottom–up processing of affective sharing, in which emotion processing brain structures play a critical role, and top–down processing in which the perceiver's motivation, intentions, and self-regulation influence the extent of an empathic experience as well as the likelihood of pro-social behavior.

As mentioned above, there is a lack of experimental studies on the potentially adverse effects of acute psychosocial stress on empathic reactions to other persons in pain. Therefore, the aim of the present study was to fill this gap by analyzing the influence of acute psychosocial stress on ratings to the observed pain in another person in an experimental setting by means of a pain paradigm (Jackson et al., 2005). Using this paradigm, Jackson et al. could show that there is a partial cerebral overlap between perceiving pain in another individual and experiencing it oneself. Moreover, the higher the activity in the dACC the higher the subjects rated the other person's pain. This points toward the role of the ACC in the affective dimension of pain, which might be specifically triggered by empathic feelings during the observation of pain in others. More importantly, in another study with the same stimulus material Jackson et al. (2006a) demonstrated that taking a third-person perspective is associated with activation of the pain-related affective neural network and led to significantly higher pain ratings than taking a non-human artificial perspective. Hence, observing pain in others as a social stimulus generates a specific mental (affective) state in the perceiver which might generate empathetic responses. Additionally, we were interested in the moderating effects of different emotion regulation skills.

Specifically, we investigated whether and in what way (1) acute psychosocial stress influences pain ratings to another person's pain, (2) acute psychosocial stress and individual differences in emotion regulation skills predict individual differences in pain ratings to another person's pain, and (3) the influence of stress on pain ratings to another person's pain is modulated by emotion regulation skills.

## Materials and methods

### Participants

The selection of participants occurred non-randomizedly (convenience sampling). Furthermore, there were few exclusion criteria for participation in the study such as health issues, language barriers, or prior experience with the Trier Social Stress Test (TSST). Two female participants had to be excluded based on psychological health issues. None had to be excluded due to language barriers or prior experience with the TSST. Thus, 120 subjects participated in the study and were randomly assigned to a Stress-Group vs. a control group (Placebo-Group). However, 14 subjects had to be excluded from the statistical analyses because the assessment of the pain ratings—our key dependent variable—failed due to technical artifacts. Furthermore, two subjects (age > 40 years) were identified as outliers with regard to their age. Out of the remaining 104 participants 52 were assigned to each group (Stress-Group: *N* = 52, age: 19–33 years, *M* = 23.95, 25 females; Placebo-Group: *N* = 52; age: 18–39, *M* = 24.15, 27 females). There were no differences between the groups regarding to age [*T*_(88)_ = 0. 263, *p* = 0. 793; please note that information about age was only available for 90 subjects] and gender [*x*^2^_(1)_ = 0.154, *p* = 0.695].

### Procedure

**Figure 1:**
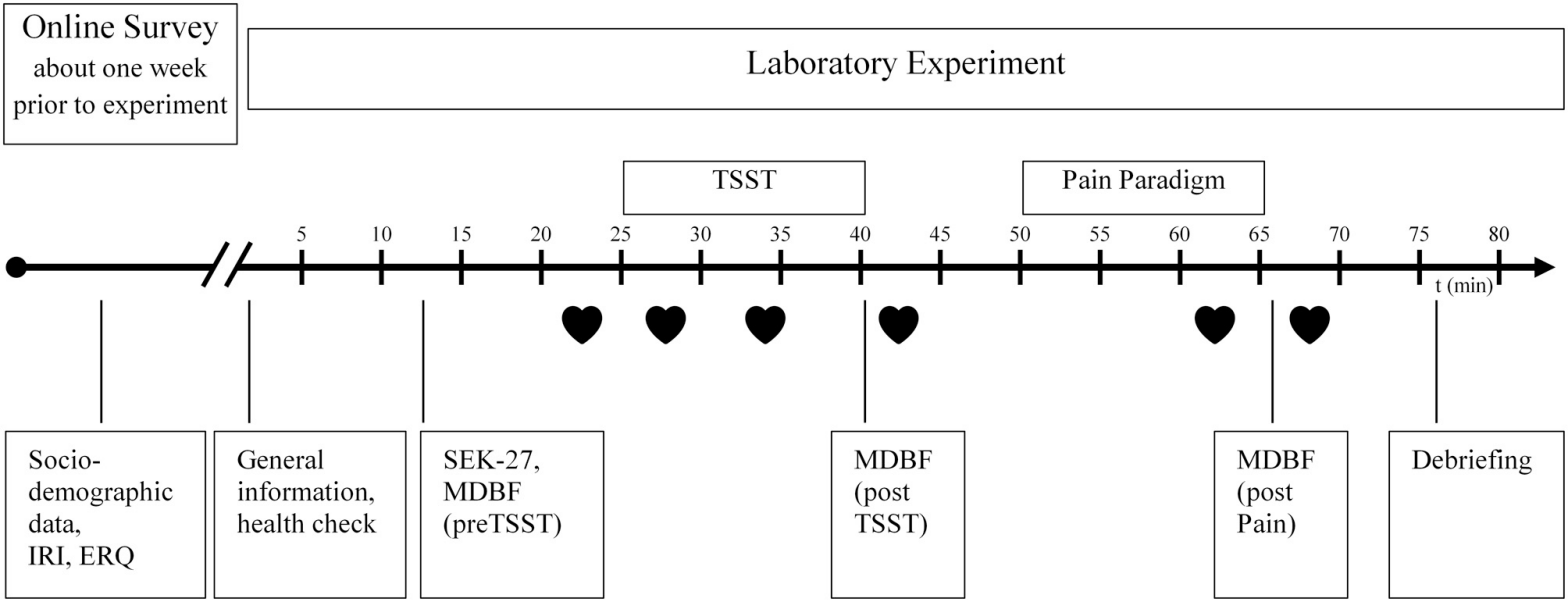
**Experimental procedure, IRI, Interpersonal Reactivity Index; ERQ, Emotion Regulation Questionnaire; SEK27, Self-Report Measure for the Assessment of Emotion Regulation Skills; MDBF, Multidimensional mood questionnaire; TSST, Trier Social Stress Test; 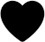, Heart rate**.

The research design was a randomized, standardized, multivariate experimental/control group comparison with pre-/post-/repeat-measurement. The participants were randomly assigned to one of the two experimental conditions [Trier Social Stress Test (TSST) = Stress-Group vs. Placebo-TSST = Placebo-Group]. A manual with a precise description of the lab procedure was developed. Two laboratory assistants were trained in the TSST and in multiple trial runs before commencing the study. The main examination lasted approximately 90 min and was conducted in the behavioral observation laboratory of the Institute for Work, Organizational, and Social Psychology at the TU Dresden. The experimental procedure is illustrated in Figure 1.

### Measurement

#### Acute psychosocial stress manipulation

The TSST was developed by Kirschbaum et al. (1993) for the analysis of stress reactions after the induction of psychosocial stress. The subtasks of the TSST are structured in such a way that threatening aspects for the social Self of an individual are included via self-involvement, social evaluation, uncontrollability and unpredictability of the examination setting (Dickerson and Kemeny, 2004). The 15-min test involves developing a speech on a preselected topic (5 min), performing the speech (5 min), and doing a mental arithmetic task (5 min). TSST tasks were performed in front of trained “evaluators,” unfamiliar to the participants. Additionally, the participants faced a camera and microphone by which they were told that their behavior will be recorded. In contrast, the Placebo-Group did not encounter judgment by an evaluator team, was not recorded, and had to speak about a topic without any self-involvement. Participants in the Placebo-Group had to prepare and then read out loud a text about a holiday trip in an empty room. Additionally, the mental arithmetic task was designed less difficult than the respective task in the Stress-Group (Het et al., 2009).

#### Cardiovascular reaction

As a psychophysiological manipulation check for the stress induction, we assessed the cardiovascular reaction. Heart rate data was continuously sampled beat-to-beat during the complete experimental procedure by the Polar © RS800cx (Polar Electro Oy, Kempele, Finland) heart rate monitor. Acceptable validity and reliability of these devices has been demonstrated (Goodie et al., 2000; Porto and Junqueira, 2009). First, we inspected heart rate data with the PolarTrainerPro5 software. Artifacts were trend corrected using the same software (moderate filter, zone = 6). Overall, the prevalence of artifacts in the individuals' complete heart rate samples was very small (*M* = 0.74%, *SD* = 2.52%). Second, we used HRV Analysis 2.0 for Windows (Niskanen et al., 2004) to calculate the mean heart rates for intervals of 5 min each. We selected the following six intervals (1) preTSST (6–1 min prior to TSST, baseline condition), (2) TSST 1 (1–6 min after starting the TSST/Placebo procedure), (3) TSST 2 (7–12 min after starting the TSST/Placebo procedure), (4) postTSST (11–16 min after TSST), (5) Pain (last 5 min of pain paradigm), and (6) postPain (1–6 min after pain paradigm). The experimental procedure is also presented in Figure 1. Because of technical problems heart rate data was eligible for *N* = 93 participants only. There was no significant difference between the groups with and without usable heart rate data concerning age (*U* = 465.50, *Z* = −0.491, *p* = 0.624), empathy for pain rating (*U* = 498.50, *Z* = −0.137, *p* = 0.891), and stress group allocation [*X*^2^_(1)_ = 0.102, *p* = 0.500]. However, there was some evidence that data dropout was higher in females than in males [*X*^2^_(1)_ = 4.981, *p* = 0.052] independent from stress manipulation. Furthermore, concerning the emotion regulation skills, modification (as measured by the SEK-27, see below) was slightly lower in the sample without heart rate data (*U* = 278.00, *Z* = −2.489, *p* = 0.013).

#### Subjective stress reaction

The MDBF (Multidimensional mood questionnaire; Steyer et al., 1997) captures the individuals' “current psychological state”—which reflects another indicator of subjective stress experience in this study. Psychological state is understood by the authors as a snap-shot of the current mood of a person which comprises three bipolar subscales of the questionnaire: “Good mood-bad mood,” “awake-tired,” and “calm-nervous.” The participant can describe his mood on a five-point scale (1 = “not at all,” 5 = “very”). In this study, participants were assessed with the MDBF on three different time points in order to assess changes of subjective stress over time (see also Figures 1, 3). In our sample, reliability coefficients of the MDBF scales proved to be high (MDBF-mood T1 α = 0.91, T2 α = 0.94, T3 α = 0.93; MDBF-alertness T1 α = 0.91, T2 α = 0.83, T3 α = 0.92; MDBF-calmness T1 α = 0.86, T2 α = 0.93, T3 α = 0.91).

#### Pain paradigm

**Figure 2:**
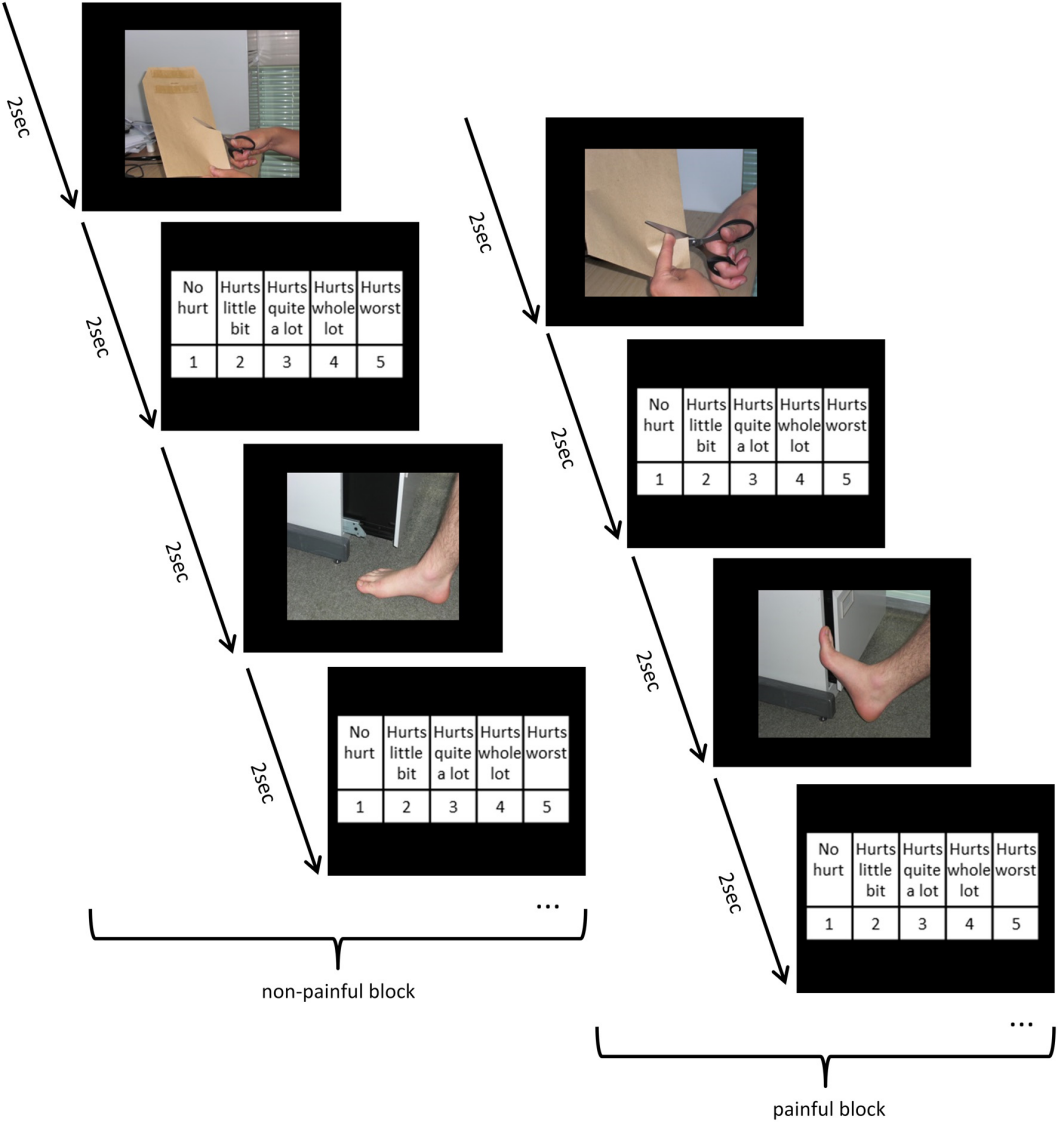
**Time flow of the pain paradigm, examples of four trials with in-house practice pictures**.

Ratings of the perceived pain in another person were measured by means of a paradigm proposed by Jackson et al. (2005). The paradigm consists of a series of 120 digital color pictures provided by Phillip Jackson and Jean Decety showing right hands and right feet in neutral, non-painful (30 pictures) and painful situations (90 pictures). The painful pictures are provided in three different pain intensities (on the basis of pain intensity ratings of 20 independent subjects; Jackson and Decety, unpublished data). Additionally we created 20 inhouse photographs for a practice trial. All pictures show common situations of everyday life with various types of pain (mechanical, thermal, and pressure-related) and have the same size (600 × 450 pixels). First, the participants were shown one practice block (20 pictures), subsequently after that they were presented with six blocks with painful images of all pain intensities and six blocks with neutral pictures (15 per block, non-painful pictures were repeatedly shown). The order of the picture blocks was randomized as well as the order of the pictures within each block. Each picture was shown for 2 s followed by a rating scale by which subjects were instructed to rate the intensity of the pain they thought the other person would experience in each situation. In order to provide pain ratings, the subjects used a 5-point Likert-scale (0 = “No hurt,” 1 = “Hurts little bit,” 2 = “Hurts quite a lot,” 3 = “Hurts whole lot,” 3 = “Hurts worst”; see Figure 2). Additionally, reaction time was recorded. By forcing the subjects to take a third-person perspective we intended to trigger empathic feelings, since empathy includes perspective taking, and Self-other distinction (Walter, 2012). Supporting this assumption, numerous studies with the same stimulus material report that perceiving and assessing painful situations in others is associated with significant changes in activity in several brain regions that are known to play a significant role in Self pain processing, which emphasizes the power of this paradigm to elicit empathic reactions (Jackson et al., 2005, 2006a,b; Moriguchi et al., 2007). Additionally, Lamm et al. (2008) report that empathic concern was considerably stronger when participants, confronted with painful facial expressions of another person, focused on the feelings of the other.

Headphones were worn by the participants in order to minimize interfering acoustic stimuli. As the instrument demands quick reactions from the participants, effects due to social desirability are minimized.

#### Emotion regulation skills

Emotion regulation skills were measured by the SEK-27 (Self-Report Measure for the Assessment of Emotion Regulation Skills). The SEK-27 was developed by Berking and Znoj (2008) with the purpose of assessing different aspects of coping with negative emotions. In the model of emotion regulation skills by Berking (2010), effective emotion regulation is conceptualized as the situation-adapted interplay of the abilities to (a) be aware of emotions, (b) identify and label emotions, (c) correctly interpret emotion-related body sensations, (d) understand external and internal prompts of emotions, (e) confront oneself with situations that cue negative emotions if necessary for attaining important goals, (f) actively modify negative emotions, (g) accept negative emotions that cannot be modified, (h) tolerate negative emotions, and (i) compassionately support oneself in distressing situations. According to tests of the model (Berking and Znoj, 2008; Berking et al., 2008), the skills of modifying negative emotions and accepting/tolerating such emotions (if they cannot be changed) may be the most essential for the maintenance and/or recovery of mental health. Each of these nine dimensions is assessed by three of the 27 items forming the scale. The scale also allows for the calculation of a summary score. The participant is to assess his coping behavior in face of negative emotions on a 5-point Likert-scale (0 = “not at all,” 1 = “rarely,” 2 = “sometimes,” 3 = “often,” 4 = “almost always”). High item values indicate high abilities and competencies. Studies on the psychometric quality of the instrument (Berking and Znoj, 2008) have shown adequate values in non-clinical (*N* = 238) populations (all α*s* 0.68–0.81 for subscales and α =0.90 for the summary scale). Reliability coefficients in our sample parallel the findings of Berking and Znoj (2008) and proved to be sufficient (awareness: α = 0.82; body sensations: α = 0.69; clarity: α = 0.77; understanding: α = 0.82; acceptance: α = 0.68; tolerance: α = 0.80; self-support: α = 0.74; readiness to confront: α = 0.80; modification: α = 0.79, general ER-skills: α = 0.81).

### Covariates

Since females showed a trend toward higher pain ratings (on painful pictures) than males [*T*_(102)_ = 1.846, *p* = 0.068], we included gender as covariate in all our analyses. Additionally, we analyzed trait empathy with the Interpersonal Reactivity Index (IRI; Davis, 1980; German Version by Paulus, 2009) and habitual emotion regulation strategies using the Emotion Regulation Questionnaire (ERQ) by Gross and John (2003; German version by Abler and Kessler, 2009). The IRI measures multiple dimensions (fantasy, perspective taking, empathic concern, and personal distress) which cover the positive and negative features of empathy. The ERQ was used in order to assess habitual preferences for two commonly applied strategies of emotion regulation: expressive suppression and cognitive reappraisal. Both, trait empathy and habitual emotion regulation strategies were included in the correlation analyses because of the association of those scales with pain ratings and several emotion regulation skills. Age was included into correlation analyses to complement sample characteristics.

### Statistical analyses

To test for differences in pain ratings and reaction times between the different pain intensities of the pictures we conducted repeated measure analyses of covariance (ANCOVA) with pain ratings or reaction times, respectively, as dependent variable, pain Intensities as repeated measures factor (non-pain pictures, pain pictures with intensity 1, pain pictures with intensity 2, pain pictures with intensity 3), Group (Stress vs. Placebo) as independent variable and gender as covariate.

For all subsequent analyses, the ratings to painful pictures were combined across the three different pain intensities resulting in two picture categories as repeated measures factor (painful and non-painful pictures).

In order to investigate whether our stress induction via the TSST was effective (dependent variables: MDBF mood, MDBF alertness, MDBF calmness, and heart rate) and to analyze the influence of stress on pain ratings (dependent variable), we used repeated measures ANCOVAs. The ANCOVAs included Group (Stress vs. Placebo) as independent variable, gender as covariate and Time (preTSST, postTSST, postPain) or Pain (non-painful pictures, painful pictures) as repeated measures factor, respectively.

To analyze associations between the covariates age, gender, trait empathy, and habitual emotion regulation, the independent variable Group (Stress vs. Placebo), as well as between the predictor emotion regulation skills and pain ratings to painful pictures, non-parametrical correlation analyses (Kendall's Tau) were performed. This step was included in order to select potential predictor variables for the following regression analyses. Variables were included when correlation coefficients were significant at least at *p* < 0.1. Correction for multiple testing was not conducted. Thus, the results should be interpreted with caution. Subsequently, we conducted a stepwise multiple regression and after that a moderated regression analysis with pain ratings on painful pictures as dependent variable, Group as independent variable and, based on the preceding correlation analysis, the selected predictor variables. All computational procedures were conducted using the PASW/SPSS Package (IBM corporation, version 19.0).

We used moderated regression analysis to test the potential moderating role of emotion regulation skills with the *Process* plugin (Hayes, 2013) for SPSS. Hence, a moderation effect would imply that the effect of acute psychosocial stress on empathy for pain depends on the level of emotion regulations skills. According to the recommendations of Aiken and West (1991) we used the following procedure for each emotion regulation skill that proved to be significantly correlated with the dependent variable (pain rating to pain pictures), and when the interaction term between acute stress induction (Group) and mean centered emotion regulation skill was significantly correlated to the pain rating. In step one, we predicted the pain rating by gender, acute psychosocial stress induction (as independent variable) and the specific emotion regulation skill (as predictor). In step two, we added the interaction term between acute psychosocial stress induction and mean centered emotion regulation skills in the multiple regression. A significant interaction term reveals moderation.

## Results

### Pain paradigm and stress manipulation check

First, we analyzed whether the participants gave pain ratings according to the pain intensities depicted in the presented stimuli. Results showed that the pain ratings increased with the presented pain intensity [Main Effect Intensities *F*_(3, 303)_ = 1.065, *p* < 0.001, η^2^ = 0.913, Interaction Intensities × Group *F*_(3, 303)_ = 1.313, *p* = 0.27, η^2^ = 0.013]. Similarly, reaction times increased from non-pain pictures, over pain pictures with intensity 1 and 2 to intensity 3 [Main Effect Intensities *F*_(3, 303)_ = 71.285, *p* < 0.001, η^2^ = 0.414, Interaction Intensities × Group *F*_(3, 303)_ = 0.374, *p* = 0.77, η^2^ = 0.004].

**Figure 3:**
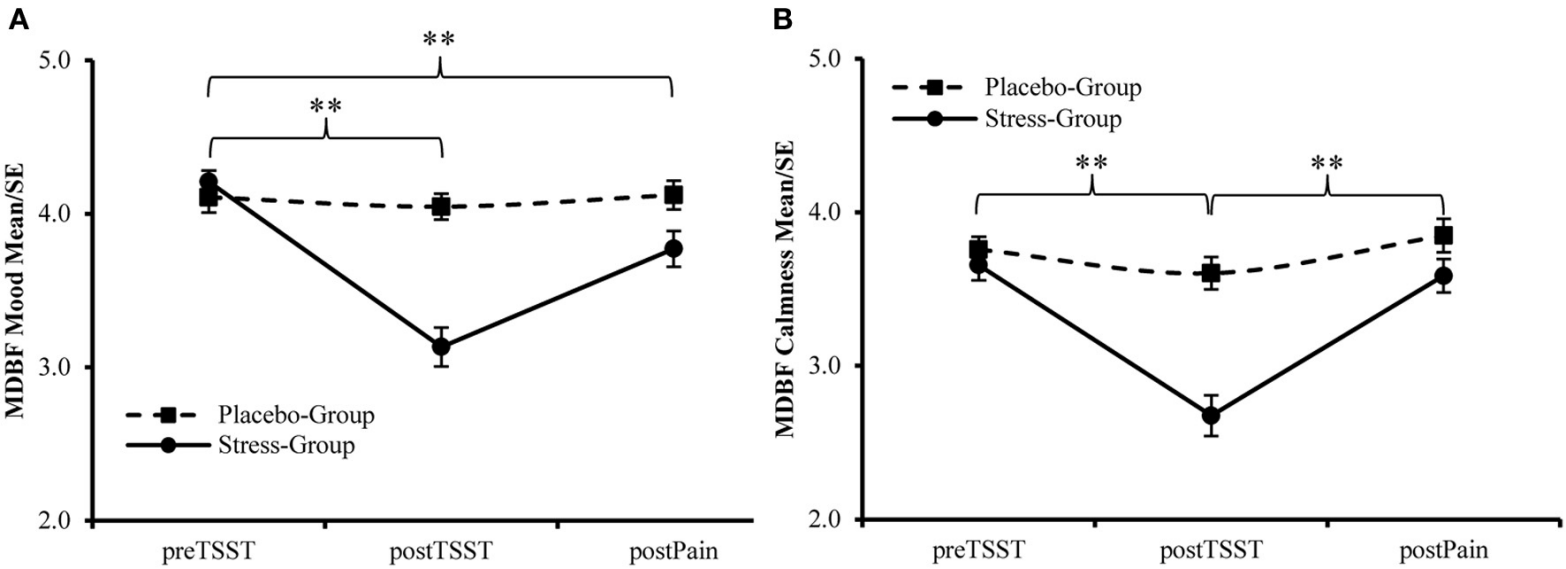
**Manipulation Check—Changes in (A) Mood and (B) Calmness during the experiment, higher values indicate a better mood and more calmness, respectively**. ^**^Interaction Effect Time × Group, *p* < 0.01.

In comparison to the Placebo-Group, the Stress-Group reported a significant reduction in mood from preTSST to postTSST [Interaction Time × Group: *F*_(1, 100)_ = 43.916, *p* < 0.001, η^2^ = 0.305] and from preTSST to postPain [Interaction Time × Group: *F*_(1, 100)_ = 11.345, *p* = 0.001, η^2^ = 0.102] as well as in calmness from preTSST to postTSST [Interaction Time × Group: *F*_(1, 102)_ = 29.854, *p* < 0.001, η^2^ = 0.230] while calmness increased again from postTSST to postPain [Interaction Time × Group: *F*_(1, 100)_ = 5.231, *p* < 0.024, η^2^ = 0.050] as measured by MDBF scales (see Figure 3). The Placebo-Group did not report any change in their mood and calmness [*F*_(1, 49)_ < 2.000, *p* > 0.10].

**Figure 4:**
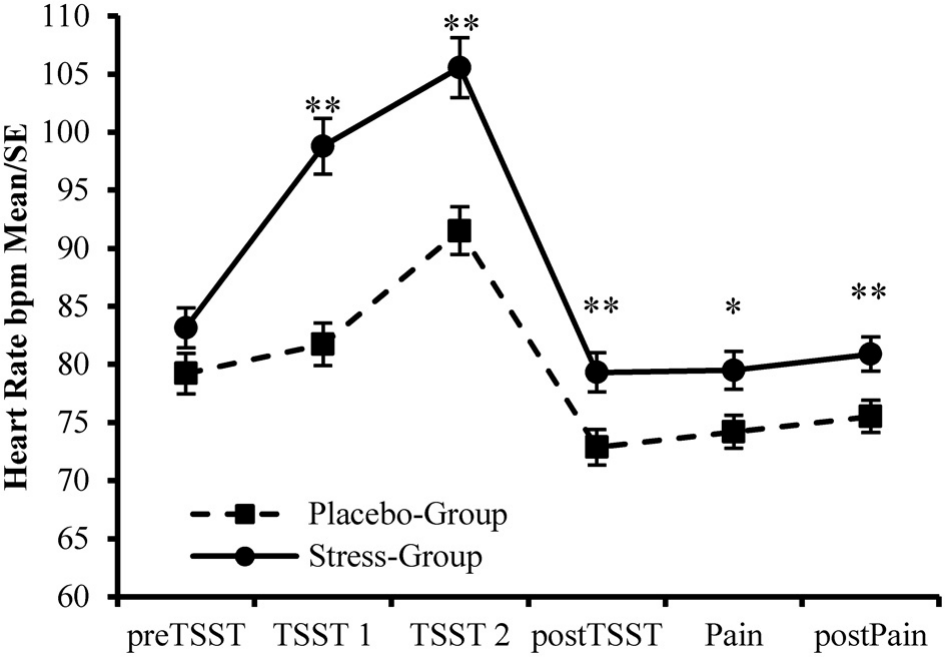
**Manipulation Check—Changes in Heart Rate during the experiment**. ^**^Between Group Effects *p* < 0.01. *Between Group Effect *p* < 0.05.

Additionally, the participants assigned to the Stress-Group showed a higher increase in heart rate during stress induction than participants in the Placebo-Group [Interaction Time × Group: *F*_(2, 178)_ = 32.739, *p* < 0.001; η^2^ = 0.269]. Afterwards values declined rapidly to stabilize during Post-TSST-procedures. However, for participants in the Stress-Group heart rate remained elevated compared to the Placebo-Group during these time points [*F*_(1, 89)_ = 7.601, *p* = 0.007, η^2^ = 0.079, see Figure 4]. This main effect was not modified by Time [*F*_(2, 178)_ = 2.617, *p* = 0.076, η^2^ = 0.029], Gender [*F*_(1, 89)_ = 0.614, *p* = 0.435, η^2^ = 0.007], or Time × Gender [*F*_(2, 178)_ = 1.069, *p* = 0.346, η^2^ = 0.012]. Furthermore, females showed a higher heart rate than males throughout the experiment [*F*_(1, 89)_ = 5.633, *p* = 0.020, η^2^ = 0.060]. This gender effect was not modified by Time [*F*_(1, 88)_ = 2.081, *p* = 0.153, η^2^ = 0.023], Group [*F*_(1, 88)_ = 1.790, *p* = 0.184, η^2^ = 0.020], or Phase [stress induction vs. pain ratings; *F*_(1, 88)_ = 0.038, *p* = 0.846, η^2^ = 0.000].

### Acute psychosocial stress and pain ratings

**Figure 5:**
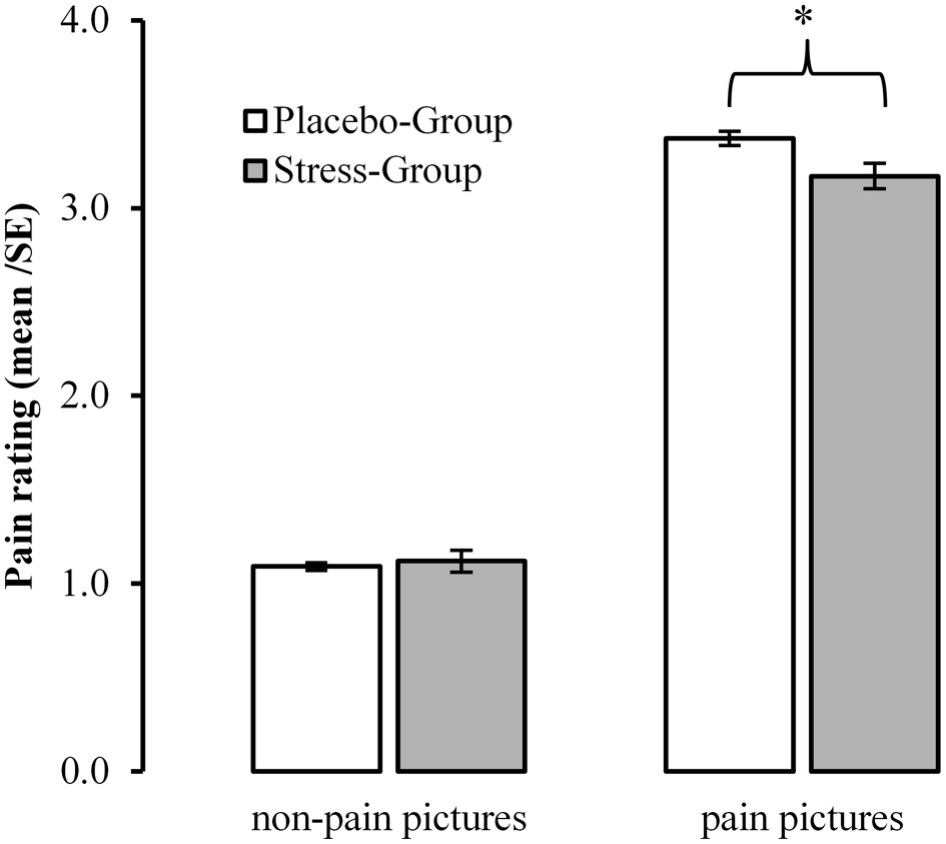
**Influences of acute psychosocial stress on ratings to pain in others**. ^*^Between Group Effect <0.05.

Whereas pain pictures were rated significantly more painful than non-painful pictures [Main Effect Pain: *F*_(1, 101)_ = 1062.235, *p* < 0.001, η^2^ = 0.913], this difference was reduced in the Stress-Group [Interaction Pain × Group: *F*_(1, 101)_ = 5291, *p* = 0.023, η^2^ = 0. 050]. *Post-hoc T*-tests revealed that participants of the Stress-Group rated the pain pictures less painful than the Placebo-Group [*T*_(102)_ = 2.280, *p* = 0.025, *d* = 0.482]. Figure 5 presents the interaction between psychosocial stress and pain judgments.

### Predictors of pain ratings

First, we used a partial correlational analysis (adjusting for gender) to examine the specific role of cardiovascular activation for the prediction of the pain ratings to pain pictures. However, individual heart rate during postTSST (*r* = −0.165, *p* = 0.117), and Pain (*r* = −0.139, *p* = 0.186) were unrelated to the pain ratings. Furthermore, the separate repetition of this analysis for both experimental subgroups (Stress vs. Placebo-Group, adjusting for gender) and both types of gender (females vs. males, adjusting for stress manipulation) revealed no significant differences in the reported intercorrelations (all *p* ≥ 0.408). Additional analyses, using the *Process* plugin (Hayes, 2013) for SPSS (5000 bias corrected bootstrapped samples, all analyses adjusted for baseline heart rate), revealed that none of the mean heart rate values for the five measurement intervals during the experiment was a mediator (simple mediation adjusting for gender, model 4 in Process) for the stress–pain judgment relation and these potential mediational effects were furthermore not moderated by gender and the measured emotion regulation skills (moderated mediation, model 7 in Process, results are not shown here).

Subsequently, to select potential predictors of the pain ratings to pain pictures in addition to psychosocial stress, we conducted a further correlational analysis. There were small, but significant by trend correlations of the pain judgments during painful situations with gender, Group (Stress vs. Placebo-Group), and the emotion regulation skill clarity (*p* < 0.10). Furthermore, we found significant correlations between pain ratings and the emotion regulation skills acceptance as well as tolerance (*p* < 0.05). For detailed correlations between the independent variable, the covariates and relevant variables with the dependent variable see Table 1.

**Table 1:** **Correlation coefficients between control variables, stress, emotion regulation skills, and pain ratings**.

	**Pain rating on painful pictures**	**1**	**2**	**3**	**4**	**5**	**6**	**7**	**8**	**9**
**SOCIODEMOGRAPHIC**										
Gender (*N* = 104)	−0.15^+^	1								
Age (*N* = 90)	0.02	−0.01	1							
**EMPATHY (TRAIT) − IRI (*N* = 102)**										
Fantasy	−0.03	−0.22^**^	0.12	1						
Perspective taking	−0.12	−0.05	−0.05	0.20^**^	1					
Empathic concern	−0.08	−0.26^**^	−0.14	0.32^**^	0.11	1				
Personal distress	0.12	−0.33^**^	0.05	0.12	−0.13	0.17^*^	1			
**HABITUAL EMOTION REGULATION − ERQ (*N* = 102)**										
Reappraisal	−0.05	−0.13	−0.07	0.03	0.25^**^	−0.02	−0.14^*^	1		
Suppression	0.12	0.28^**^	0.10	−0.20^**^	−0.04	−0.30^**^	−0.17	0.05	1	
Stress vs. Placebo (*N* = 104)	−0.15^+^	0.03	−0.06	−0.10	0.10	0.02	−0.00	0.01	−0.10	1
**EMOTION REGULATION SKILLS − SEK27 (*N* = 104)**										
Awareness	0.02	−0.32^**^	−0.06	0.11	0.11	0.22^**^	0.06	0.08	−0.20^**^	0.03
Body sensations	−0.03	−0.16^+^	−0.23^**^	0.07	0.14	0.13	0.05	0.09	−0.15^**^	0.06
Clarity	−0.13^+^	−0.14	−0.21^**^	0.07	0.14	0.04	−0.06	0.09	−0.21^**^	0.03
Understanding	−0.06	−0.12	−0.20^*^	0.10	0.14	0.06	−0.00	0.00	−0.24^**^	−0.07
Modification	−0.07	0.05	−0.05	−0.03	0.25^**^	−0.02	−0.18^*^	0.25^**^	−0.01	−0.00
Acceptance	−0.17^*^	−0.01	−0.17^*^	0.08	0.18^*^	0.06	−0.19^**^	0.11	−0.16^*^	−0.04
Tolerance	−0.15^*^	0.12	−0.09	−0.00	0.25^**^	−0.05	−0.26^**^	0.14^*^	−0.00	−0.01
Self-support	−0.02	−0.09	−0.10	0.05	0.24^**^	−0.01	−0.14	0.39^**^	−0.04	0.01
Readiness to confront	0.05	−0.13	−0.09	−0.05	0.11	0.00	−0.16^*^	0.30^**^	0.01	0.00

* *p < 0.05*,

** *p < 0.01*,

+ p < 0.10 (two-tailed); IRI, Interpersonal Reactivity Index; ERQ, Emotion Regulation Questionnaire; SEK, Self-Report Measure for the Assessment of Emotion Regulation Skills.

Subsequently, based on the intercorrelations (Table 1) the variables mentioned above (gender, Group and emotion regulation skills as predictors of the pain ratings on painful pictures) were included in a stepwise multiple regression analysis. In the first block, gender was included as covariate since it showed a significant (although small) intercorrelation with the pain ratings to painful pictures. This resulted in a beta weight of ß = −0.180 (*p* = 0.068) for gender and the model was significant with 2% explained variance (*R*^2^ = 0.023). In the second block, the experimental manipulation of acute psychosocial stress (Stress-Group vs. Placebo-Group) was included in the statistical prediction. This resulted in a significant beta weight of ß = −0.214 (*p* = 0.028) for the experimental induction of stress. The model summary shows that there is a significant increase (*p* < 0.01) in explained variance from model 1 (2% explained variance) to model 2 (6% explained variance). In the third step, emotion regulation skills (clarity, acceptance, and tolerance) were included in the regression. Out of the selected emotion regulation skills only acceptance was included in the prediction model ß = −0.231 (*p* = 0.015). This was associated with a further significant increase in explained variance of the pain ratings by 4–10% (*N* = 104, *p* < 0.01). The remaining emotion regulation competencies are excluded in the course of the stepwise regression analysis. In the following, we examined a further potential model for the stress–pain rating relation: The moderating impact of emotion regulation skills.

### Emotion regulation skills modulate the relation between acute psychosocial stress and ratings of pain in others

Firstly, we computed interaction terms between Group and the mean centered value for each emotion regulation skill (SEK27) and included these terms in the correlation analysis. This resulted in small, but significant correlations of the pain ratings on painful pictures with Group × clarity (*r* = −0.162, *p* = 0.028) and Group × tolerance (*r* = −0.162, *p* = 0.026), and to a trend toward significant correlations with Group × understanding (*r* = −0.138, *p* = 0.060) and Group × acceptance (*r* = −0.140, *p* = 0.055). Hence, we added these interaction terms into the following moderated regression analysis.

**Figure 6:**
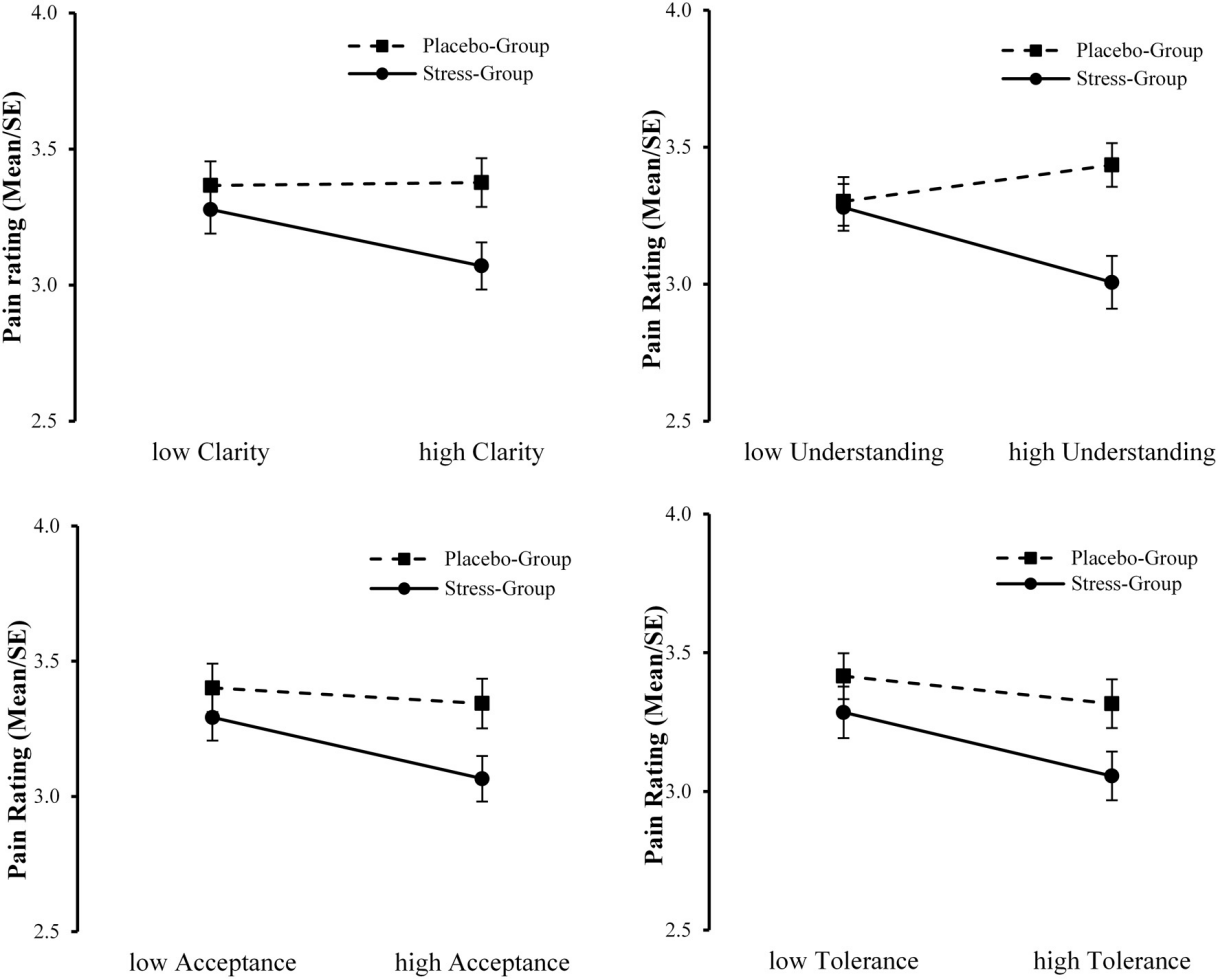
**Interaction plots of Group with clarity, understanding, acceptance, and tolerance on ratings to pain in others**.

Secondly, we conducted a moderated regression analysis with the covariate gender, Group as independent variable, emotion regulation skills as predictors (clarity, understanding, acceptance, tolerance), and with the respective interaction terms between group and emotion regulation skill. Our assumption concerning the potential moderating effect of specific emotion regulation skills on the relation between acute psychosocial stress and pain ratings had to be rejected (*N* = 104). None of the tested interaction effects were significant (Group × clarity: *B* = −0.176, *SE* = 0.145, *p* = 0.228; Group × understanding: *B* = −0.175, *SE* = 0.121, *p* = 0.151; Group × acceptance: *B* = −0.095, *SE* = 0.123, *p* = 0.442). However, one effect showed a trend toward significance (Group × tolerance: *B* = −0.192, *SE* = 0.110, *p* = 0.083). In Figure 6 the interactions of Group with the investigated emotion regulation skills on the pain ratings on painful pictures are illustrated.

## Discussion

The aim of the present study was to analyze the influence of acute psychosocial stress and emotion regulation skills on the judgments of another person's pain—an indicator of empathic feelings. We found that after inducing psychosocial stress, participants rated pictures of others in painful situations significantly less painful than participants that did not undergo a stress induction. In addition to the effect of stress induction, individual differences in the ability to regulate one's own emotions by acceptance predicted the judgment of pain in others. To be specific, subjects that reported a higher acceptance rated other people's pain as significantly lower. Moreover, our results further suggest that the ability to tolerate negative emotions modulated the association between stress and pain ratings.

In this final section, the findings of the experimental study are summarized and discussed. Firstly, we will discuss the influence of acute psychosocial stress on the appraisal of pain others and how this relates to empathic processing. Subsequently, we integrate and interpret the findings of the regression and moderation analyses. Finally, research limitations are discussed and suggestions for further research are provided.

### The appraisal of pain in others and acute psychosocial stress

In the present study, we attempted answering the question whether the experience of psychosocial stress influences the empathic reaction to perceived pain in others. Referring to prior research (Smeets et al., 2009; Newton, 2013), we assumed that individuals experiencing a social stress induction show changes in their pain ratings when confronted with another person's pain in a standardized empathy for pain paradigm (Jackson et al., 2005). Although the participants in our study did not view faces expressing pain, which has been shown to instigate strong empathic reactions (Craig et al., 2010), but hands and feet under painful stimulation, they judged the pain of the depicted person in accordance to the presented pain intensity which is mirrored in significant differences in reaction times. This replicates findings by Jackson et al. (2005, 2006a) who report higher pain ratings on another person's pain to painful as compared to neutral pictures. Jackson et al. (2006a) also found that pain ratings were lower and reaction times higher when the subjects were taking the other-perspective as compared to a self-perspective, indicating that this requires an additional frame of reference, which is necessary for empathic behavior. Since we adopted the other-perspective instruction from the study by Jackson et al., we consider the measurement of pain ratings to another person's pain applied in our study suitable for the assessment of empathy for pain (see also Jackson et al., 2006b).

Furthermore, our study demonstrates that participants who had been exposed to acute psychosocial stress, and accordingly showed higher ratings concerning their subjective stress experience as well as elevated physiological responses, revealed significantly lower values in their pain ratings. This effect could not be observed in participants assigned to the non-stress control condition.

It has been shown that negative emotions increase pain unpleasantness ratings on own pain (Villemure et al., 2003; Rainville et al., 2005; Loggia et al., 2008a,b), which seems to be modulated by the dACC (Villemure and Bushnell, 2009). Moreover, Loggia et al. (2008a) demonstrated that a state of high empathy (i.e., a positive affective link with another) was related to higher intensity and unpleasantness ratings of own pain experiences while observing the other person receiving similar painful stimulation. In contrast, in our study the experience of psychosocial stress, which is accompanied by negative mood, led to a reduction in intensity ratings of pain in others.

There are several factors that might contribute to the differences between our findings and those of the cited studies. First, almost all of the studies mentioned above did not investigate the observation of pain in others. Second, they reported results on unpleasantness ratings while we found effects on intensity ratings which might tap into different processes. Finally, we did not observe any association between mood and pain ratings. Hence, the effect of psychosocial stress on the intensity ratings of another person's pain may constitute a specific empathy related process, which is independent of the emotional state of the Self. Consistent with our results, a study by Guo et al. (2013) demonstrated that short-term media violence exposure reduced pain ratings and the activation of AI and aMCC on pictures showing fingers and ears in painful situations. The authors interpreted their findings as a process of physiological desensitization, while one may also conclude that watching violent films induces a physiological stress reaction (Weidmann et al., 2009; Hasan et al., 2013) that may account for the effects on pain empathy.

Research considering the effects of stress on empathy predominantly focuses on problems associated with chronic stress in health care employees (Koehl-Hackert et al., 2012; West, 2012). Especially the negative consequences of stress have been intensively investigated. However, the respective studies exclusively focus on *chronic* stress as predictor for risky states such as burnout or lacking empathy as well as physical and mental strain (Schaufeli et al., 2009; Newton, 2013). The consequences of *acute* stress for empathy have not been scientifically investigated so far, although an experimental manipulation of acute stress could allow for better opportunities to test the effect of potential modulating variables.

The results of our study show that acute stress significantly impacts the appraisal of pain in another person, which can be interpreted as an effect on empathic feelings. Although the reductions in pain ratings in the stressed as compared to the non-stressed group are comparatively small, they are particularly relevant insofar as the stress induction was only short-termed as confirmed by psychological and physiological measurements. Thus, our results strongly confirm prior findings on consequences of chronic stress using a cost-efficient experimental setting to test the relations between acute psychosocial stress and empathy for pain. We also assume that the contribution of the predictors (emotion regulation skills acceptance and tolerance) to individual differences in in the pain ratings remained low, because we used an acute stress paradigm to induce stress reactions in our participants. As compared to chronic stress experiences that last for months or even years, acute psychosocial stress is not only much shorter, but also better manageable for most subjects.

It could be argued that the reducing effect of stress on the appraisal of pain in others may be explained by the detrimental influences of stress on PFC functions, which have been shown to impair social cognition (Smeets et al., 2009). However, in this experimental setting we are not able to evaluate whether the reduction in the empathic reaction to pain allows for a negative interpretation. Alternatively, our data might also be interpreted in terms of adaptive strategies. If you are under stress, reducing your own negative feelings in spite of observing and understanding (in terms of cognitive empathy) the negative feelings of another person might be of great importance, since it has been discussed that empathic concern and not personal distress leads to helping behavior (Batson et al., 2003; Decety, 2011; Newton, 2013). Accordingly, the ability to regulate emotions ought to be of predictive value for the extent of the empathic feelings and behavior.

### Predictors of the appraisal of pain in others

Our next question concerned the potential role of acute psychosocial stress and specifically associated emotion regulation skills as predictors of the pain ratings, with emotion regulation skills being conceptualized as prolonged state (current state including the previous week). By means of a stepwise multiple regression analysis, the impact of the potential predictors was tested. Overall, 10% of the variance in the ratings to pain in others was explained by (objective) acute stress and the emotion regulation skill acceptance. In detail, acute psychosocial stress reduced the pain ratings, as already discussed above. In addition, the higher participants rated their ability to use acceptance as emotion regulation strategy, the lower they rated pain in others.

Kohl et al. (2012) conclude from their meta-analytic review that acceptance strategies proved to be superior to other emotion regulation strategies (e.g., suppression, distraction, reappraisal) with respect to pain tolerance. Additionally, investigations using the fear-avoidance-endurance-model in pain therapy (Hasenbring et al., 2009) emphasize that the acceptance of an unpleasant situation is an important resource in the course of coping with pain (McCracken and Eccleston, 2003). Similarly, acceptance might aid persons in observing others being in pain to reduce empathic distress. In support of this, Newton (2013) points out that learning to regulate affective empathic responses might help health care professionals to establish a certain degree of empathic detachment which in turn permits them to provide objective care. Hence, the application of emotion regulation in order to down regulate one's own empathic reaction (in response to negative emotions of others) may be essential, but only if effective strategies are being applied. For example, dysfunctional habitual emotion regulation is thought to be a central feature of psychopathology (Abler et al., 2007, 2010; Berking and Wupperman, 2012) whereas acceptance may be a particularly promising strategy for long-duration stressors (Braams et al., 2012). To support this, Berking et al. (2012) found that acceptance is beneficial to mental health regardless of its potential to facilitate the modification of (negative) emotions.

Following this, to extend our findings on the direct effects of stress and emotion regulation, we will subsequently discuss our results on indirect effects. Thereby, we will consider interactions between stress and emotion regulation skills with regard to their impact on the appraisal of pain in others, which might provide additional implications for pain empathy.

Decety (2011) emphasizes that stress might have detrimental effects on empathic reactions, insofar as observing pain in others may also constitute a threat to the individual that can lead to personal distress (Yamada and Decety, 2009). This association might even be more pronounced in individuals exhibiting dysfunctional emotion regulation strategies. If not regulated, this distress can conflict with the observer's capacity to be of assistance to the other. Following this model, we assumed that the influence of acute stress on pain ratings to another person's pain would be modulated by the ability to regulate one's own emotions. The results of the moderated regression analysis point to an interaction between the induction of acute stress and the emotion regulation skill tolerance. Specifically, subjects that experienced stress revealed reduced ratings of pain in others, but this association was more pronounced in subjects that showed a high ability to tolerate negative emotions. Additionally, the results descriptively imply an interaction between stress and the ability to understand the prompts of emotions (see Figure 6). This would also implicate that participants with a high ability in understanding their own feelings show lower pain ratings (implicating lower empathic distress) but only under stress. The perception of pain in others triggers bottom-up processes of affective sharing which results in a negative emotion (Decety and Meyer, 2008). However, participants that previously underwent a stress induction already experienced negative feelings and (additionally) had to regulate their empathic reaction in order to prevent empathic distress.

Our results further strengthen the importance of acceptance/tolerance as emotion regulation strategies which refuse to focus on altering one's negative emotions. Strikingly, one of the two psychopathology-related features of reappraisal is termed emotional resistance or not-acceptance of emotional events (Werner and Gross, 2010). In contrast, acceptance and tolerance of negative emotions prevent dysfunctional regulation attempts that would judge one's internal experience as unacceptable and suppress the emotional response. Acceptance and tolerance as rather functional regulation strategies would allow for more flexible responses. It is therefore not surprising that acceptance of one's internal experience is proved to be an adaptive strategy for working with one's emotional responding (Hayes et al., 2006; Dalrymple and Herbert, 2007; Valdivia-Salas et al., 2010).

## Limitations

As mentioned above, the contribution of the predictors to the pain ratings is comparably low, which is probably due to the application of an acute stress paradigm. We also assume that the time between stress induction and pain measurements was too long to observe more profound effects. Future studies should prefer conducting an empathy paradigm during or shortly after stress induction. Above, potential variables that might be of further predictive value like, for example, current chronic stress, development of empathic behavior, depression, anxiety, or emotional repertoire (see also De Vignemont and Singer, 2006) were not measured. Furthermore, heart rate was not measured trial-by-trial in the pain paradigm, which would have provided an objective measure to substantiate the pain ratings.

Another limitation of the empathy for pain paradigm used in our study is that it merely consists of pictures showing hand and feet under painful stimulation. An empathy paradigm in which the subjects are confronted with more emotional cues such as movements or gestures and facial expressions might have resulted in higher emotional responses. Another limitation may concern the reference point within the pain ratings which might differ between the participants: It was left up to the participants whether they related the task to an episode of pain experienced at present, or to anticipated pain occurring in the future.

It has been shown that women show stronger reactions in emotion studies (Domes et al., 2010; Whittle et al., 2011) as well as investigations into empathy (Han et al., 2008; Proverbio et al., 2009). Although we consider the inclusion of women as well as men in our study as strength of the design this might have reduced possible effects of stress on pain ratings.

In view of the experimental setting applied in this study, we assume a high internal validity of the analysis resulting in a low likelihood of alternative explanations for the findings demonstrated here. By the randomized assignment of the participants to the experimental conditions individual confounding factors are controlled for. In addition, further potential confounders (e.g., health) were eliminated by taking additional measures prior to randomization.

A further limitation results from the sampling method applied here: We used a convenience sample of students taking part voluntarily rather than a probabilistic sample. In addition, participants were aware of taking part in a scientific study, which may have been associated with several behavioral changes. Finally, our sample was very homogenous concerning age and education (i.e., largely psychology students). This could have limited the variance in stress reaction and empathy for pain.

## Conclusion

To our knowledge, the present study is the first that experimentally supports a direct effect of acute psychosocial stress on reactions to pain in others, drawing from an interdisciplinary scholarly perspective on stress, emotion and social (neuro-) science. Moreover, our results emphasize the important role of functional emotion regulation for a healthy reaction to other peoples' pain and, hence, for empathy for pain (i.e., how an “observer” understands the pain of a “sufferer;” Singer et al., 2004). Empathy describes an emotional reaction to the observed behavior and feelings of another person and we might show in our study that under stress this reaction might be altered. However, our findings that certain emotion regulation strategies modulate these stress effects, offers the opportunity for interventions. Findings from research on mindfulness suggest that participation in mindfulness-based stress reduction programs (MBSR) is associated with changes in the concentration of gray matter in brain regions involved in emotion regulation, self-referential processing, and perspective taking (Holzel et al., 2011), which are essential for empathic processing. In addition, Berking et al. (2010) demonstrated that a manualized emotion-regulation training (Integrative Training of Emotional Competencies; iTEC; Berking, 2010) can improve emotion regulation skills of police officers. All these results additionally emphasize the interplay between emotion regulation strategies and susceptibility to stress.

It has also been discussed that empathic sharing of negative feelings might raise the vulnerability to stress and negative emotions. Thus, by comparing empathy training with compassion training it could be shown that compassion is crucial in counteracting the activation of negative emotions (Klimecki et al., 2013a,b). Additionally, compassion training has proved effective even in short-term versions (Leiberg et al., 2011). This implicates that humans possess adaptive regulation strategies that go beyond reappraisal and suppression. Following this, drawing attention to the creation of positive situations that come along with positive feelings, such as joy, optimism, pride and serenity, constitutes a resource. Only if people possess sufficient resources relevant actions, for example altruistic behavior, can evolve.

Underestimations of the experienced pain intensity in another person by a caregiver or significant other carry the risk of the person in pain feeling misunderstood or, more importantly, the risk of increasing the physiological harm to that person (Hadjistavropoulos and Craig, 2002). A biopsychosocial perspective on pain has therefore been postulated as necessary for research and practice if care is to be effectively delivered to individuals in need (Hadjistavropoulos et al., 2011). This perspective includes that not only the pain expression but rather the characteristics of the observer and contextual variations are also important (e.g., Decety and Jackson, 2004; Goubert et al., 2005). Our study clearly supports this view. It seems obvious that under stress, empathic reactions to pain in others may result in pro-social behavior at best, but without regulatory strategies social behavior will not occur.

### Conflict of interest statement

The authors declare that the research was conducted in the absence of any commercial or financial relationships that could be construed as a potential conflict of interest.
